# Oral administration of the *Aureobasidium pullulans*-derived β-glucan effectively prevents the development of high fat diet-induced fatty liver in mice

**DOI:** 10.1038/srep10457

**Published:** 2015-07-16

**Authors:** Shiho Aoki, Atsushi Iwai, Koji Kawata, Daisuke Muramatsu, Hirofumi Uchiyama, Mitsuyasu Okabe, Masahiro Ikesue, Naoyoshi Maeda, Toshimitsu Uede

**Affiliations:** 1 Aureo Science Co., Ltd., Sapporo, Hokkaido, Japan; 2 Aureo Co., Ltd., Kimitsu, Chiba, Japan; 3 Division of Molecular Immunology, Institute for Genetic Medicine, Hokkaido University, Sapporo, Japan; 4 Department of Matrix Medicine, Institute for Genetic Medicine, Hokkaido University, Sapporo, Japan

## Abstract

*Aureobasidium pullulans*-derived β-glucan (AP-PG) consisting of a β-(1,3)-linked glucose main chain and β-(1,6)-linked glucose branches is taken as a supplement to improve health. This study demonstrates that oral administration of AP-PG is effective to prevent the development of high-fat diet (HFD)-induced fatty liver in mice. Here, C57BL/6N mice were fed with a normal diet or HFD, and AP-PG diluted in drinking water was administered orally. After 16 weeks, the serological analysis showed that HFD-induced high blood cholesterol and triglyceride levels were reduced by the oral administration of AP-PG. Further, HFD induced-fatty liver was significantly reduced by the oral administration of AP-PG. The triglyceride accumulation in the liver was also significantly reduced in mice administered AP-PG. Liver injury as indicated by an increase in serum alanine aminotransferase (ALT) in the HFD-fed mice was significantly reduced in the mice administered AP-PG orally, and the gene expression of cholesterol 7 alpha-hydroxylase (CYP7A1) which is known to be involved in cholesterol degradation in the liver was significantly increased in the AP-PG administered mice. These results suggest the possibility that the oral administration of AP-PG is effective to prevent the development of non-alcoholic fatty liver disease (NAFLD).

Non-alcoholic fatty liver disease (NAFLD) is known to be a major health problem in many developed countries. The NAFLD is closely associated with obesity and type 2 diabetes^1^,^2^, and is known to be a major risk factor for cardiovascular diseases^3^. The NAFLD is mainly caused by excess energy intake, and improvements of dietary habits are assumed to be the first choice for prevention of NAFLD. However, it is often difficult to alter food habits and lifestyles. Therefore, a supplement could be an alternative means for the prevention of fatty liver, and some food derivatives, such as whey protein^4^,^5^, soybean protein^6^,^7^, and anthocyanins^8^, effective for the prevention of HFD-induced fatty liver have been reported.

Black yeast, *Aureobasidium pullulans* is widely used in industrial production of pullulan by fermentation^9^. Further, *A. pullulans* extracellularly produces soluble β-glucan consisting of a β-(1,3)-linked glucose main chain and β-(1,6)-linked glucose branches under specific growth conditions^10^,^11^. The *A. pullulans*-cultured fluid containing the β-glucan as a main component exhibits immune stimulatory activity, and is consumed as a supplement in many countries, as well as *A. pullulans*-cultured fluid is believed to exhibit beneficial effects in delaying the onset a number of diseases, and has been reported to exhibit anti-tumor^12^,^13^, anti-allergy^14^, and also anti-infectious disease^15^,^16^,^17^ activities in mouse models.

In the present study, we demonstrate that development of HFD-induced fatty liver is reduced by oral administration of purified *A. pullulans*-derived β-glucan (AP-PG) in mice. The HFD-induced high blood cholesterol and triglyceride levels were significantly reduced in the mice orally administered with AP-PG. Oral administration of AP-PG significantly reduced HFD-induced adipocyte hypertrophy in epididymal white adipose tissue. Further, HFD-induced fatty liver was significantly reduced in the mice which had been subject to orally administered AP-PG. Importantly, liver injury as indicated by an increase in serum alanine aminotransferase (ALT) in HFD-fed mice was significantly reduced by orally administered AP-PG, and the gene expression of cholesterol 7 alpha-hydroxylase (CYP7A1) which is known to be involved in cholesterol degradation in the liver was significantly increased in AP-PG administered mice. These results show the potential for β-glucan produced by *A. pullulans* as a supplemental food in the prevention of diseases caused by high fat diet life styles.

## Results

### Effects of orally administered AP-PG on lipid metabolism with HFD feeding

**Figure 1:**
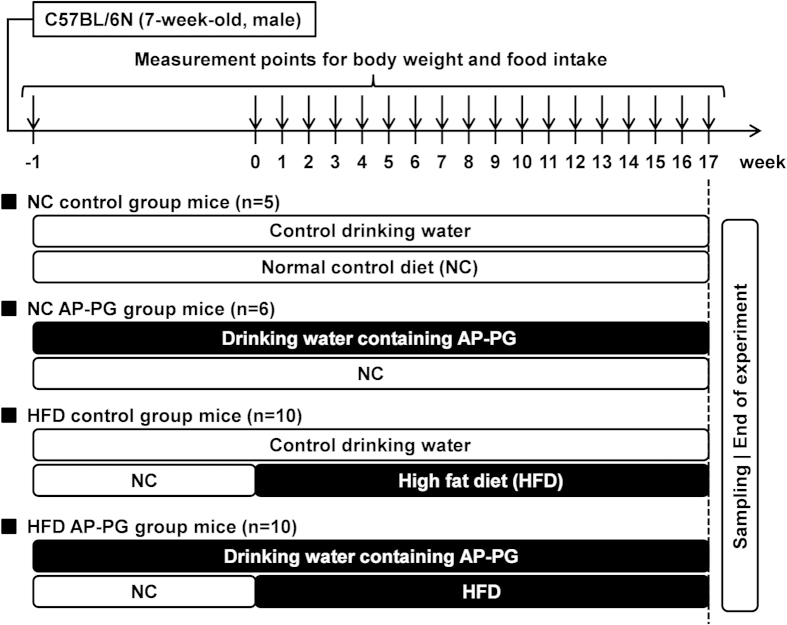
Experimental design of the study.

**Figure 2:**
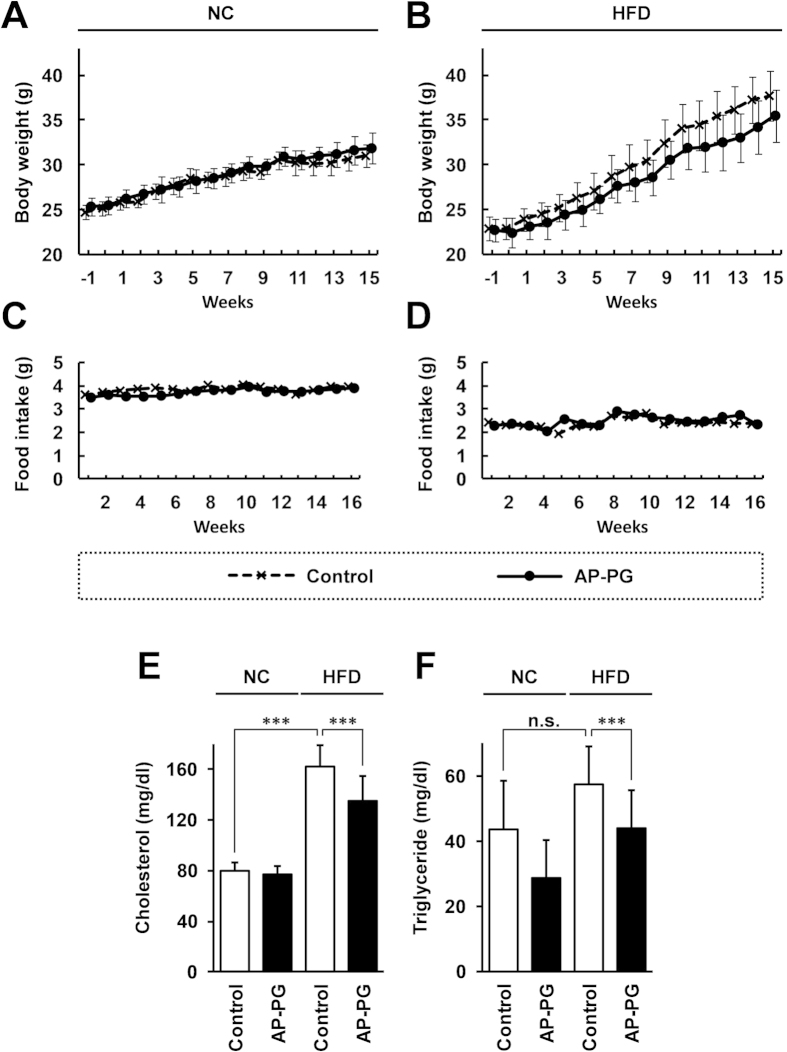
Effect of AP-PG on body weight changes, and on plasma levels of cholesterol and triglyceride (**A**–**D**) The body weight and food intake of each group of mice were monitored weekly. (**E**,**F**) After the period of the experiment, plasma levels of cholesterol and triglyceride in the non fasted mice were measured as described in Methods section. Error bars indicate standard deviations. NC: normal control diet. HFD: high-fat diet. Triple asterisks (***) indicates statistically significant differences (p < 0.005). n.s.: not significant.

To evaluate the effects of AP-PG on lipid metabolism with HFD, we used a mouse model, and monitored the body weight and performed serological analysis. The experimental design of this study is shown in Fig. 1. The C57BL6/N mice were separated into 4 groups: (1) mice receiving normal control diet (NC) and the control drinking water (n = 5, NC control group); (2) mice receiving NC and the drinking water containing AP-PG (n = 6, NC AP-PG group); (3) mice receiving the high-fat diet (HFD) and the control drinking water (n = 10, HFD control group); and (4) mice receiving the HFD and the drinking water containing AP-PG (n = 10, HFD AP-PG group). The preparation of the control drinking water was described in Materials and Methods. The mice were pre-administered with AP-PG for 1 week, subsequently the mice received HFD for 16 weeks, and the administration of AP-PG was continued until the end of the experiment. The body weight and food intake of the mice are shown in Fig. 2A-D. The body weight and food intakes of the AP-PG and control group mice were similar. In HFD administered mice, the mean body weight of AP-PG group mice at the late phase of the experiment was 2 to 3 g lower than that of the control group mice (difference not statistically significant). Therefore, it was concluded that the orally administered AP-PG does not exhibit a significant effect on the HFD-induced increment in body weight.

Next, to evaluate the effect of orally administered AP-PG on the blood cholesterol and triglyceride levels, a serological analysis was performed. As shown in Fig. 2E and F, the high blood cholesterol and triglyceride levels induced by HFD were significantly reduced by the oral administration of AP-PG. These data suggest the possibility that orally administered AP-PG is effective in the improvement of the lipid metabolism.

### Effect of orally administered AP-PG on adipocyte hypertrophy in epididymal white adipose tissue

**Figure 3:**
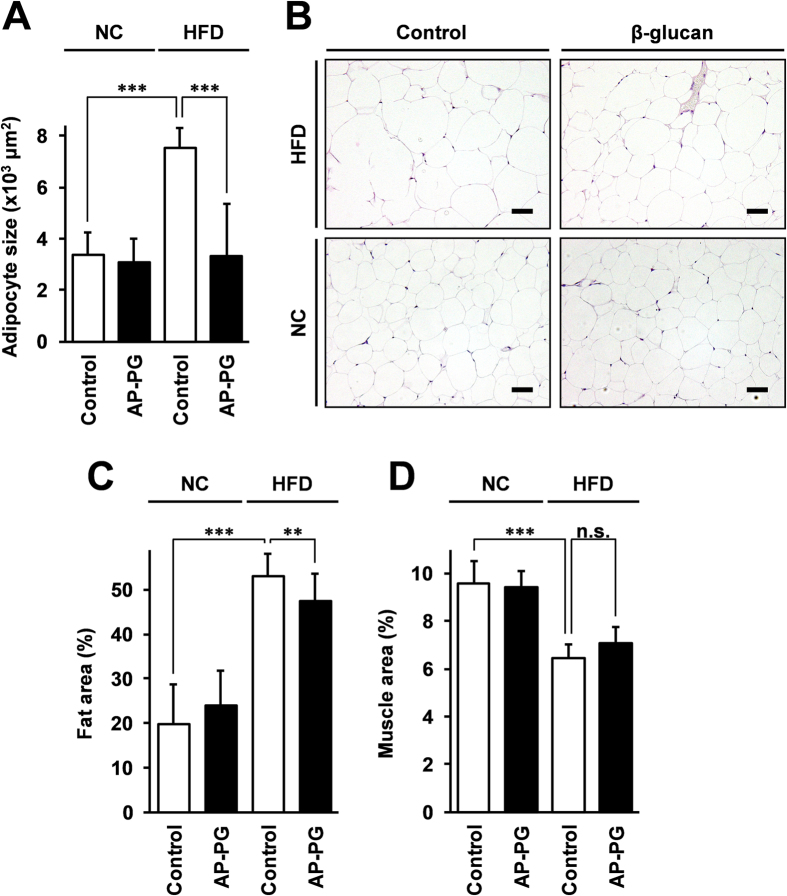
Oral administration of AP-PG is effective to suppress hypertrophy of white adipocytes caused by high-fat diet. (**A**) After the period of the experiment, epididymal white adipose tissue was isolated from the mice, and stained with hematoxylin and eosin. Subsequently, the diameter of adipocytes in the epididymal white adipose tissue of the mice were measured by image analysis using HE stained sections. (**B**) Histological photomicrograph of HE stained sections. Scale bars: 50 μm. (**C**, **D**) Fat (**C**) and muscle (**D**) areas in the epididymal white adipose tissue of the mice analyzed by CT scan analysis. Error bars indicate standard deviations. NC: normal control diet. HFD: high-fat diet. Double asterisks (**) and triple asterisks (***) indicate statistically significant differences (p < 0.01 and p < 0.005 respectively). n.s.: not significant.

We next examined whether orally administered β-glucan has any effect on the accumulation of lipids in adipose tissue. Epididymal white adipose tissue was isolated from the mice, and HE-stained paraffin sections were examined microscopically, followed by image analysis. As shown in Fig. 3A, the HFD-induced increment in the adipocyte size was significantly reduced by oral administration of AP-PG, a microphotograph of the histology shown in Fig. 3B. Further, image analysis of CT scans demonstrated that the fat area around the epididymal white adipose tissue was significantly reduced in the HFD AP-PG group mice when compared with that in the HFD control group mice (Fig. 3C). At the same time, the muscle area around the epididymal white adipose tissue was not statistically significantly different in the HFD AP-PG administered and HFD control groups. These results suggest that the oral administration of AP-PG is effective in the reduction of HFD-induced adipocyte hypertrophy.

### Oral administration of AP-PG is effective in reduction of HFD-induced development of fatty liver

**Figure 4:**
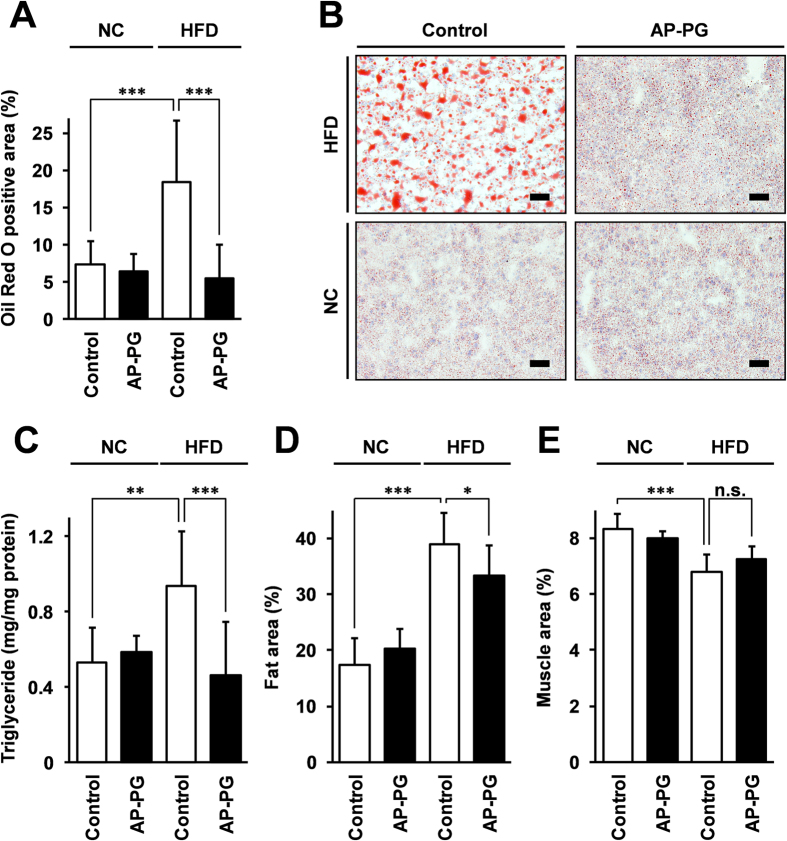
Oral administration of AP-PG is effective to prevent development of fatty liver induced by high-fat diet. (**A**) After the period of the experiment, liver tissue was isolated from the mice, and the liver sections were stained with Oil O Red. Subsequently, Oil O Red-positive areas in the liver sections were measured by image analysis. (**B**) Microphotograph of Oil O Red stained sections. Scale bars: 50 μm. (**C**) Measurements of accumulation of triglyceride in the liver of the non-fasted mice. After the period of the experiment, triglyceride in the whole liver was measured as described in Methods section. NC: normal control diet. HFD: high-fat diet. Single asterisk (*), double asterisks (**), and triple asterisks (***) indicate statistically significant differences (p < 0.05, p < 0.01, and p < 0.005 respectively). n.s.: not significant.

To evaluate the effect of the orally administered AP-PG on the development of HFD-induced fatty liver, sections of livers isolated from the mice were stained with Oil O Red and analyzed microscopically. The Oil O Red positive area was statistically significantly reduced in the HFD AP-PG group mice when compared with the HFD control mice (Fig. 4A). It must be noted that the Oil O Red positive area in the sections of liver of the HFD AP-PG group mice was reduced at a level comparable to that in the NC-fed mice group, as suggested in the histology microphotograph in Fig. 4B.

The fatty liver is at least partially, if not all caused by abnormal accumulations of triglyceride in the liver, and the amount of triglyceride in the livers isolated from the mice was quantified. The results show that the concentration of triglyceride per protein of the liver was significantly reduced in the HFD AP-PG group mice when compared with the HFD control group mice (Fig. 4C). In addition, the CT scans show that the fat area in the liver was significantly reduced in the HFD AP-PG group mice when compared with that in the HFD control group mice (Fig. 4D). The muscle areas of the HFD control and HFD AP-PG groups were comparable. These results demonstrate that oral administration of AP-PG is effective in reducing HFD-induced fatty liver in mice.

### Oral administration of AP-PG increases the expression of cholesterol metabolism-related genes in the liver

**Figure 5:**
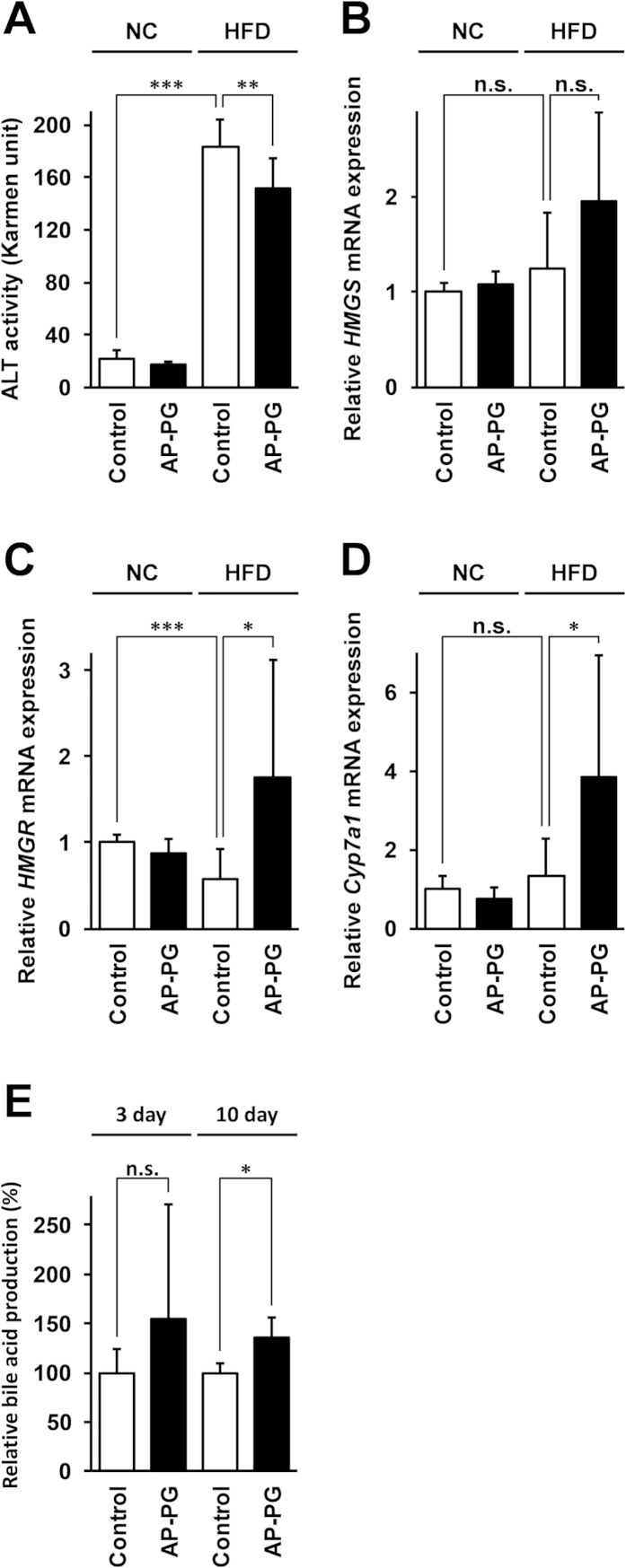
Effects of oral administration of AP-PG on liver injury caused by HFD (A) ALT activity in the serum of the mice.(**B**-**D**) Expression of Hydroxymethylglutaryl-CoA (HMG-CoA) reductase (HMGR; **B**), HMG-CoA synthase (HMGS; **C**), and cholesterol 7 alpha-hydroxylase (CYP7A1; **D**) mRNAs in the liver quantified using real-time RT-PCR. Data represent relative expression values compared with the NC control after normalization with glyceraldehyde 3-phosphate dehydrogenase (GAPDH) mRNA expression. (**E**) Feces were collected from mice at the time point indicated in the Figure, and the amounts of fecal bile acid were measured as described in Method section. Data represent relative bile acid amounts compared with the control. Error bars indicate standard deviations. NC: normal control diet. HFD: high-fat diet. Single asterisk (*), double asterisks (**), and triple asterisks (***) indicate statistically significant differences (p < 0.05, p < 0.01, and p < 0.005 respectively). n.s.: not significant.

It is known that abnormal accumulations of triglyceride in the liver cause hepatocellular injury. Alanine aminotransferase (ALT; also known as GPT [glutamic pyruvic transaminase]) is an enzyme where the serum level is elevated by hepatocellular injury^18^,^19^,^20^. To determine this for the present study, the effects of orally administered AP-PG to protect HFD-induced liver injury were investigated by monitoring serum ALT levels. As shown in Fig. 5A, the serum ATL levels in the HFD control group were significantly lower than those in the HFD AP-PG group.

Cholesterol is synthesized from the acetyl coenzyme A (acetyl Co-A), and is degraded into bile acids in the liver. Hydroxymethylglutaryl-CoA (HMG-CoA) reductase (HMGR) and cholesterol 7 alpha-hydroxylase (CYP7A1) are known to be rate-limiting enzymes in cholesterol synthesis and cholesterol degradation, respectively. To determine this here, we investigated the mRNA expressions of these genes in the liver using real-time RT-PCR, and in addition to these genes, the expression of another gene that is important for the cholesterol synthesis, the HMG-CoA synthase (HMGS) gene, was also investigated. The results show that although the mean of HMGS gene expression in the HFD AP-PG group mice was slightly higher than that in the HFD control mice, the difference was not statistically significant (Fig. 5B). However, the gene expressions of HMGR and CYP7A1 in the liver were significantly increased in the HFD AP-PG group mice when compared with the expression in the HFD control group mice (Fig. 5C and D, respectively).

Previous report demonstrated that the transgenic mice overexpressing CYP7A1 gene in liver are resistant to HFD-induced fatty liver^21^. Further, the CYP7A1 transgenic mice indicate the phenotype of which cholesterol synthesis in liver is activated and triglyceride synthesis is decreased^22^. Therefore, increment of CYP7A1 expression after oral administration of AP-PG is thought to be crucial for prevention of development of fatty liver, the disease caused by excess triglyceride accumulation in liver. To confirm CYP7A1 activity is increased in the liver after oral administration of AP-PG, the amounts of fecal bile acids were monitored. The bile acids synthesized from cholesterol in liver are secreted into the lumen of the intestine. In addition, CYP7A1 is known to be the rate limiting enzyme for the cholesterol degradation and also for the bile acid synthesis. The results show that amount of fecal bile acids was significantly increased in AP-PG group mice after 10 days (Fig. 5E). These results suggest that orally administered AP-PG could play a role to induce CYP7A1 in liver.

## Discussion

In this study it is clearly demonstrated that orally administrated AP-PG is effective in reducing HFD-induced high serum cholesterol and triglyceride, HFD-induced adipose tissue hypertrophy, HFD-induced liver injury as indicated by the elevation of ALT, and HFD-induced fatty liver (accumulation of lipid in liver cells). More importantly, we found that oral administration of AP-PG led to a significant up-regulation of HMGR and CYP7A1, rate-limiting enzymes for cholesterol synthesis and cholesterol degradation, respectively. Especially, up-regulation of CYP7A1 mRNA expression could be the molecular basis for the improvement of the fatty liver, liver injury, and cholesterol levels indicated in this study. As mentioned in the results section, the transgenic mice overexpressing CYP7A1 gene in liver are resistant to HFD-induced fatty liver and indicate the phenotype of which cholesterol synthesis in liver is activated and triglyceride synthesis is decreased^21^,^22^. On the other hand, the HMGR inhibitors, statins are widely used for the treatment of disorder of energy utilization and storage (metabolic syndrome) including NAFLD^23^. Thus, the up-regulation of HMGR mRNA expression is not always thought to be beneficial effect to prevention of HFD-induced fatty liver. The increment of HMGR mRNA expression in liver found in this study would be caused by the results of up-regulation of CYP7A1 expression as similar with that in the CYP7A1 overexpressing mice. The response to HFD in CYP7A1 overexpressing mice is resemble with the mice orally administered AP-PG. For instance, the HFD-induced increment in the adipocyte size is significantly reduced in the CYP7A1 overexpressing mice^21^. Therefore, the several beneficial effects of orally administered AP-PG shown in this study might be closely related in the induction of CYP7A1 expression. The CYP7A1 gene expression is negatively regulated by the activation of c-Jun N-terminal kinase (JNK), the stress-activated protein kinase^23^ and that JNK activation was suppressed by β-glucan, derived from a mushroom, *Ganoderma lucidum*^24^,^25^. This may suggest that immunomodulatory activity of AP-PG is related in the up-regulation of CYP7A1 gene expression.

However, it must be pointed out that β-glucans including AP-PG are known as immune stimulators, and also to be a dietary fiber. Dietary fibers are thought to be effective to inhibit excess absorption of lipids in the small intestine. Therefore, it is possible that the effect of AP-PG for the prevention of development of fatty liver demonstrated in this study is mainly depending on the function of AP-PG as a dietary fiber. To rule out this possibility, we investigated effect of orally administered AP-PG on cholesterol absorption in the small intestine using radioisotope-labeled cholesterol. Here it was found that the absorption of radioisotope-labeled cholesterol was not statistically significantly different in AP-PG-administered and control mice (Supplemental Figure S1). Thus our data strongly suggest that the effect of AP-PG as a dietary fiber may not significantly affect the results obtained in this study.

The results of cholesterol absorption experiment suggest that immune stimulatory activity of AP-PG would be related in the effects of orally administered AP-PG for the prevention of HFD-induced fatty liver. However, how the immune stimulatory activity of AP-PG exhibits preventive effect to the HFD-induced fatty liver is unknown. An inflammatory cytokine, IL-6 is known to be involved in the regulation of lipid metabolism. The mice lacking IL-6 gene are known to be developed mature-onset obesity^26^. In addition, previous report demonstrated that treatment with IL-6 alleviates HFD-induced fatty liver and normalizes the serum ALT level using mouse and rat models^27^,^28^. We previously demonstrated that the expression of IL-6 mRNA is increased after treatment with AP-PG in macrophage-like cell line, RAW264.7 cells^11^. Therefore, there is a possibility that induction of IL-6 after stimulation with AP-PG is related in the effect of AP-PG for the prevention of HFD-induced fatty liver.

Several supplements, such as whey protein^4^,^5^, soy been protein^6^,^7^, and anthocyanins^8^ are known to exhibit beneficial effects to prevent HFD-induced fatty liver. These supplements are thought to exhibit beneficial effects to prevent HFD-induced fatty liver through the antioxidant activity. Previous report showed that *A. pullulans* derived β-glucan indicates weak antioxidant activity^29^. However, as mentioned above, β-glucan is known to be a dietary fiber. β-glucans including AP-PG are not able to absorb in small intestine of human, like as these other supplements. Therefore, the effects of AP-PG to prevent development of HFD-induced fatty liver are thought to be indirect manner through the activation of intestinal immunity, and the mechanism to exhibit the effects of orally administered AP-PG for the prevention of HFD-induced fatty liver development would be distinct with these other supplements.

In this study, we demonstrated that oral administration of AP-PG is effective to prevent development of HFD-induced fatty liver in mice. In addition, the absence of significant negative side effects of AP-PG on the lipid metabolism in the NC-fed mice is noteworthy. This would indicate the possibility that AP-PG could be a safe supplemental food supplement. Although a human intervention study is required for the substantiation, these results suggest the possibility that the *A. pullulans*-cultured fluid containing β-glucan has beneficial effects as a supplement for prevention of NAFLD caused by excess energy intake. Further investigations are required to establish the efficacy of AP-PG for the prevention of NAFLD as a supplement for humans.

## Methods

### Mice

Specific pathogen-free C57BL/6N (8 weeks old) male mice were purchased from CLEA Japan (Tokyo, Japan). HFD containing 0.15% cholesterol and 21% milk fat was purchased commercially available product (57BD; TestDiet, Richmond, IN) and used in this study. All animal experiments were performed in accordance with the guidelines of the Bioscience Committee of Hokkaido University and were approved by the Animal Care and Use Committee of Hokkaido University.

### Preparation of *Aureobasidium pullulans-*derived β-glucan

Culture condition of *A. pullulans* for production of β-glucan by fermentation is described elsewhere^16^. For preparation of the *A. pullulans*-derived β-glucan (AP-PG), *A. pullulans*-cultured fluid was subjected to diatomaceous earth filtration to remove cell debris, subsequently low molecular weight components were removed by ultrafiltration with a cut-off molecular weight of 20,000 (Q2000; Advantec, Tokyo, Japan). Subsequently, the β-glucan was precipitated with ethanol at the concentration of 80%, and used for the study. The purity of the prepared β-glucan was estimated to be better than 99% using high performance liquid chromatography (HPLC). The low molecular weight fraction filtrated by the ultrafiltration was used for the control in this study. The removal of β-glucan in the control fraction was confirmed using HPLC, and the contamination of β-glucan was estimated to be less than 1%.

### Measurement of cholesterol and triglyceride levels

Concentrations of cholesterol and triglyceride in non-fasted serum were measured using commercially available kits (Triglyceride E-test Kit and Cholesterol E-test Kit; Wako Pure Chemical, Osaka, Japan) according to the manufacturer’s protocols. For the quantification of the concentration of triglyceride in the liver of non-fasted mice, approximately 50 mg of frozen liver sample was homogenized in 1 ml of SET buffer (250 mM Sucrose, 2 mM EDTA, and 10 mM Tris-HCl [pH 7.4]) using 5.0 mm diameter glass beads with agitation at 3200 rpm for 30 sec. After two freeze-thaw cycles, the homogenates were passed through a 27-gauge syringe needle three times, and then subjected to a single freeze-thaw cycle. Concentrations of triglyceride and protein in the liver homogenates were measured using a Triglyceride E-test Kit (Wako Pure Chemical) and a Pierce BCA protein assay kit (Thermo scientific, Rockford, IL) respectively in accordance with the manufacturers’ protocols.

### Measurement of ALT activity in serum

Measurement of alanine aminotransferase (ALT) activity in mouse serum was performed using a transaminase CII-test Wako kit (Wako Pure Chemical) according to the manufacturer’s protocols.

### Real time RT-PCR analysis

The total RNA extractions from the mouse livers were performed using TRIzol reagent (Invitrogen, Carlsbad, CA), and cDNA was synthesized from the total RNA by reverse transcription using Transcriptor Universal cDNA Master (Roche, Indianapolis, IN). Real-time PCR to monitor the mRNA expression was carried out using Fast Start DNA Master PLUS SYBR Green I (Roche), and measured by a Light Cycler (Reche). Each procedure was performed in accordance with the manufacturer’s protocol. The following specific primer sets for Hydroxymethylglutaryl-CoA (HMG-CoA) reductase (HMGR), HMG-CoA synthase (HMGS), and cholesterol 7 alpha-hydroxylase 1 (CYP7A1) were used in this study: HMGR (sense primer: 5’- TTGCTACTTCTGCGAAGGCA -3’, anti-sense primer: 5’- TCCGTGAGGGAATTCAAGGC -3’); HMGS (sense primer: 5’- AAGCTCAGAGAGGACACCCAT -3’, anti-sense primer: 5’- CGAGCGTAAGTTCTTCTGTGC -3’); and CYP7A1 (sense primer: 5’- TTGCTACTTCTGCGAAGGCA -3’, anti-sense primer: 5’- TCCGTGAGGGAATTCAAGGC -3’).

### Image analysis

The Computed Tomography (CT) scan and analysis of CT images were performed using an Latheta LCT-200 CT (Aloka, Tokyo, Japan). The analysis of the histochemical images was performed using Adobe Photoshop (Adobe Systems, San Jose, CA).

### Statistical analysis

To check for significant differences between the indicated pairs of data, a two-tailed unpaired Student’s t-test was performed in this study.

## Additional Information

**How to cite this article**: Aoki, S. *et al*. Oral administration of the *Aureobasidium pullulans*-derived ß-glucan effectively prevents the development of high fat diet-induced fatty liver in mice. *Sci. Rep.* **5**, 10457; doi: 10.1038/srep10457 (2015).

## Supplementary Material

Supporting Information
