# Supplementary material for: Oral administration of the Aureobasidium pullulans-derived β-glucan effectively prevents the development of high fat diet-induced fatty liver in mice
Source: Sci Rep. 2015 Jul 16;5:10457. doi: 10.1038/srep10457 (PMC4503978; doi:10.1038/srep10457)
Supplement: Supporting Information [file srep10457-s1.pdf]

Supplemental information

Oral administration of the β-glucan produced by *Aureobasidium pullulans* effectively prevents the development of high fat diet-induced fatty liver in mice.

Shiho Aoki [1], Atsushi Iwai [1, 2*], Koji Kawata [1, 2], Daisuke Muramatsu [1], Hirohumi Uchiyama [2], Mitsuyasu Okabe [2], Masahiro Ikesue [3], Naoyoshi Maeda [3], and Toshimitsu Uede [3, 4].

[1] Aureo Science Co., Ltd., Sapporo, Hokkaido, Japan; [2] Aureo Co., Ltd., Kimitsu, Chiba, Japan; [3] Division of Molecular Immunology and [4] Department of Matrix Medicine, Institute for Genetic Medicine, Institute for Genetic Medicine, Hokkaido University, Sapporo, Japan.

*** Correspondence should be addressed to Atsushi Iwai, Aureo Science Co., Ltd., Hokudai Business Spring, North 21, West 12, Kita-ku, Sapporo, Hokkaido, 001-0021, Japan; Phone: +81-11-757-1316; Fax: +81-11-738-1317; E-Mail: iwai-atsushi@aureo.co.jp**

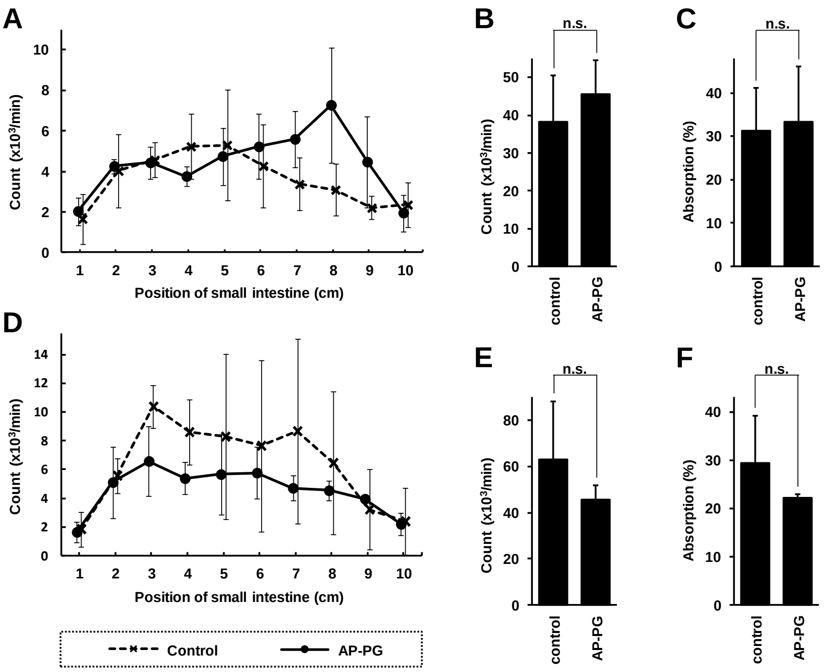

Supplementary Figure S1: Effects of orally administered AP-PG on cholesterol absorption in small intestine.

(A-C) C57BL/6J mice were orally administered with AP-PG diluted in drinking water at a concentration of 100 mg/ml for 7 days. After fasting 16 hrs, the mice were orally administered with 200 μl corn oil (SIGMA) containing ^{14}C-labeled cholesterol (40μCi/ml; Perkin elmer, Waltham, MA). (D-E) C57BL6J mice were orally administered with the β-glucan (2 mg/ml, 0.2 ml/mouse) through a syringe fitted with a ball-type feeding needle. Subsequently, the mice were orally administered with corn oil containing ^{14}C-labeled cholesterol. After 2 hrs, these mice were sacrificed, and small intestine was isolated from the mice. The isolated small intestine was washed with PBS and sliced into the 2 cm pieces. The sliced small intestines were dissolved using 1 ml of Soluene-350 (Perkin elmer) and added 5 ml of liquid scintillation cocktail (AQUASOL-2; Perkin elmer). The absorption of radiolabeled cholesterol was measured using a liquid scintillation counter. Control group mice were administered with β-glucan-removed *Aureobasidium pullulans*-cultured fluid by ultrafiltration. Data represent count of each position of small intestine (A, D), count of whole small intestine (B, E), and percent absorption in whole small intestine (C, F). The percent absorption was calculated using following formula; percent absorption = count of whole small intestine / (count of administered cholesterol - count remaining in stomach) x100. Error bars indicate standard deviation (n=3). n.s.: not significant.
